# Overuse of the hip external rotators: greater trochanter apophysitis in the karate kid

**DOI:** 10.1590/0100-3984.2017.0065

**Published:** 2018

**Authors:** Rafael Seiji Kubo, Eduardo Noda Kihara Filho, Eduardo Kaiser U. N. Fonseca, Adham do Amaral e Castro, Durval do Carmo Barros Santos

**Affiliations:** 1 Imaging Department, Hospital Israelita Albert Einstein, São Paulo, SP, Brazil.

*Dear Editor,*

**Figure 1:**
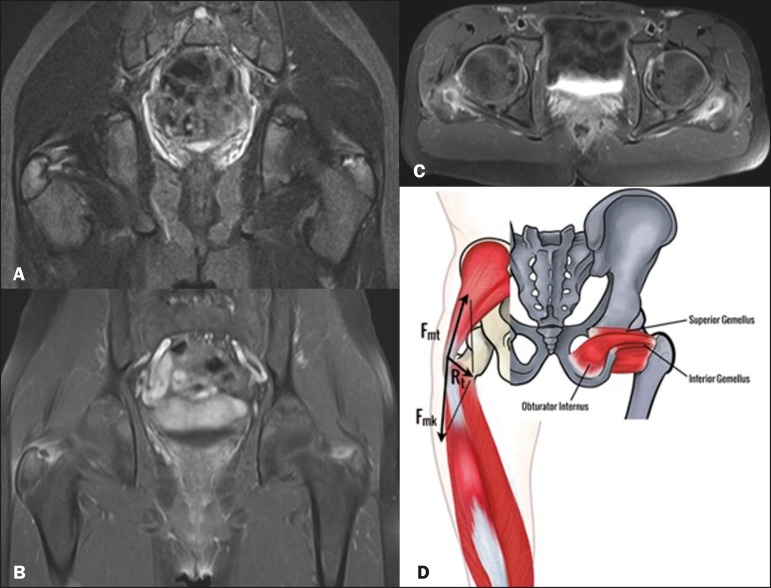
Coronal STIR weighted (A) and contrast-enhanced coronal (B) and axial (C) fast spin-echo T1-weighted magnetic resonance imaging scan of the hip showing external rotators insertional tendinopathy, peritendinitis and irregularities in both trochanters, with small avulsed cortical fragments and enhancement. Schematic representation of the obturator internus, gemellus superior, and gemellus inferior muscles inserting into the greater trochanter and the tension band mechanism, the sum of vector forces in the greater trochanter being nearly perpendicular to the apophyseal plate (D).

A 13-year-old male presented to our institution with an approximately one-month history of pain in both hips that had worsened in the last two weeks, after a soccer match. There was no definitive history of trauma. The patient was a young athlete who practiced soccer and martial arts (karate) regularly. On physical examination, there was tenderness in both hips, with pain that radiated to both thighs and diminished with rest. An X-ray of the pelvis was taken in the emergency department to rule out fractures. The X-ray showed mild irregularity and sclerosis of both greater trochanters. It was also possible to see small peritrochanteric bone fragments. After a few days, the patient underwent a magnetic resonance imaging scan, which showed insertional tendinopathy and peritendinitis in the obturator internus, gemellus superior, and gemellus inferior muscles (external rotators), bilaterally. There were also irregularities in both greater trochanters, as well as small avulsed cortical fragments with intense bone edema and enhancement (Figure 1A-C). After this initial investigation, clinical and imaging findings suggested bilateral traction apophysitis. Treatment consisted in non-operative management, with good outcome. Clinical follow-up showed good recovery, with complete resolution of the symptoms.

Apophyses are sites of tendon attachment. In children and adolescents, they are approximately 2-5 times weaker than are the surrounding structures. The greater trochanter apophysis develops by the age of two years and is expected to close at approximately 16 years of age^(1)^.

The apophyses can be injured by acute forces. The chronic mechanism may also occur in a context of repetitive and strong load forces, notably in young athletes, which is known as traction apophysitis/osteochondrosis^(2)^. On X-ray, apophysitis usually manifests as widening of the apophysis and subchondral sclerosis. These findings are diagnostic in an appropriate clinical context^(1)^.

In most cases, apophysitis occurs at typical sites, such as the tibial tubercle (Osgood-Schlatter disease), distal patella (Sinding-Larsen-Johansson disease), and iliac crest (superior or inferior). Because of normal biomechanical forces, apophysitis of the greater trochanter is very rare, only a few cases having been reported, most of them secondary to gluteal muscle traction^(3-8)^.

The double tension band, a mechanism postulated by Heimkes et al.^(6)^, as depicted in Figure 1D, would explain the greater trochanter stability, and thus the rarity of apophysitis at this site. It is based on opposition forces of the gluteus medius and gluteus minimus muscles, as well as those of the counteracting knee extensors (mainly the vastus lateralis muscle). The sum of the vector forces in the greater trochanter would be nearly perpendicular to the apophyseal plate and works as an active stabilizer.

During the practice of karate, the mechanism of external rotation is frequently called upon. The mains muscles used are the gluteus medius, gluteus minimus, semitendinosus, and semimembranosus. During external rotation of the hip, the obturator internus, gemellus superior, and gemellus inferior muscles contract and their vector forces are parallel to the apophysis plate, with no distraction forces applied to the physes (Figure 1D). This mechanism explains why there is no physeal enlargement in external rotator overuse. In conclusion, the greater trochanter is a rare site of apophysitis of which that the radiologist must be aware.
